# Supplementary material for: Hepcidin Protects Yellow Catfish (Pelteobagrus fulvidraco) against Aeromonas veronii-Induced Ascites Disease by Regulating Iron Metabolism
Source: Antibiotics (Basel). 2021 Jul 12;10(7):848. doi: 10.3390/antibiotics10070848 (PMC8300743; doi:10.3390/antibiotics10070848)
Supplement: Supplementary file 1 [file antibiotics-10-00848-s001.zip › antibiotics-1266074-supplementary.pdf]

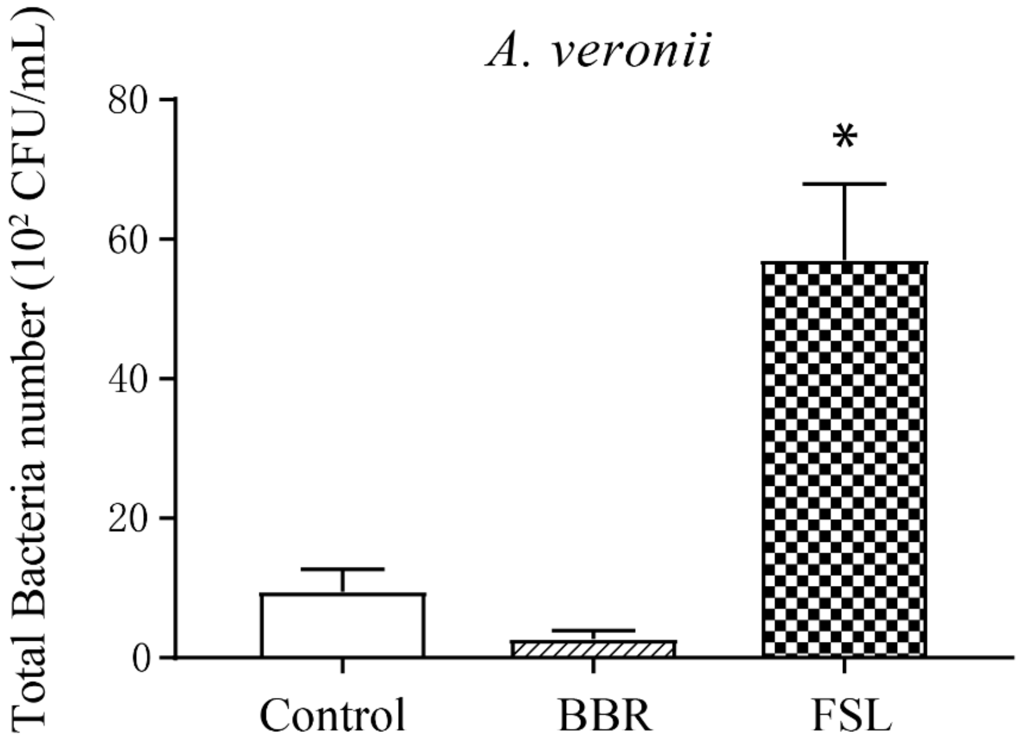

Figure S1. Bacterial counting after 6-hour incubation of BBR or FSL with hepatocytes followed by 3-hour infection with *A. veronii.*
